# Are the Main Methionine Sources Equivalent? A Focus on DL-Methionine and DL-Methionine Hydroxy Analog Reveals Differences on Rainbow Trout Hepatic Cell Lines Functions

**DOI:** 10.3390/ijms23062935

**Published:** 2022-03-08

**Authors:** Karine Pinel, Cécile Heraud, Guillaume Morin, Karine Dias, Annaëlle Marcé, Linda Beauclair, Stéphanie Fontagné-Dicharry, Karthik Masagounder, Martina Klünemann, Iban Seiliez, Florian Beaumatin

**Affiliations:** 1 Université de Pau et des Pays de l’Adour, E2S UPPA, INRAE, NUMEA, 64310 Saint-Pée-sur-Nivelle, France; cecile.heraud@inrae.fr (C.H.); guillaume.morin@inrae.fr (G.M.); karine.dias@inrae.fr (K.D.); annaellemarce@gmail.com (A.M.); linda.beauclair@inrae.fr (L.B.); stephanie.fontagne-dicharry@inrae.fr (S.F.-D.); iban.seiliez@inrae.fr (I.S.); 2 Evonik Operations GmbH, Rodenbacher Chaussee 4, 4D-63457 Hanau, Germany; karthik.masagounder@evonik.com (K.M.); martina.kluenemann@evonik.com (M.K.)

**Keywords:** aquaculture, nutrition, rainbow trout, cell lines, metabolism, methionine

## Abstract

The replacement of fishmeal by plant proteins in aquafeeds imposes the use of synthetic methionine (MET) sources to balance the amino acid composition of alternative diets and so to meet the metabolic needs of fish of agronomic interest such as rainbow trout (RT-*Oncorhynchus mykiss*). Nonetheless, debates still exist to determine if one MET source is more efficiently used than another by fish. To address this question, the use of fish cell lines appeared a convenient strategy, since it allowed to perfectly control cell growing conditions notably by fully depleting MET from the media and studying which MET source is capable to restore cell growth/proliferation and metabolism when supplemented back. Thus, results of cell proliferation assays, Western blots, RT-qPCR and liquid chromatography analyses from two RT liver-derived cell lines revealed a better absorption and metabolization of DL-MET than DL-Methionine Hydroxy Analog (MHA) with the activation of the mechanistic Target Of Rapamycin (mTOR) pathway for DL-MET and the activation of integrated stress response (ISR) pathway for MHA. Altogether, the results clearly allow to conclude that both synthetic MET sources are not biologically equivalent, suggesting similar in vivo effects in RT liver and, therefore, questioning the MHA efficiencies in other RT tissues.

## 1. Introduction

By 2050, the global population will reach 9 billion humans. Hence, providing a sustainable food supply to that exponentially growing human population is one of the core challenges for the future. Aquaculture can definitely play an important role in fulfilling this goal. Indeed, over the last four decades, aquaculture production has multiplied by five to finally provide more than half of the fish consumed worldwide in 2014 [1]. Nonetheless, and despite the intrinsic capacities of aquaculture farmers and industries to sustain and improve production levels, new economic and ecological issues have arisen. One of these problems, related to the increase demand for fish feeds, is the soaring prices of fishmeal (FM), which is still considered the most nutritious and digestible ingredient for farmed fish. Of particular concern are salmonids such as rainbow trout, which use a balanced set of amino acids for protein synthesis as other species but are adapted to use amino acids (AAs) as their preferred energy source over carbohydrates (as most carnivorous fish). Therefore, they require high levels of dietary proteins (35–45% of their meal) that can no longer be supplied by FM due to their limited availability and high demand. Multiple strategies are currently developed to replace FM by other protein sources, among which vegetable meals (VM) appear as the most convenient. Although, nowadays, VM are replacing FM in larger portions in fish diets, a 100% VM diet does not allow a normal and healthy growth of fish mainly because plant proteins display an imbalanced AA composition. Notably, the essential amino acid methionine (MET) is under-represented in VM compared to FM. Therefore, covering fish metabolic needs by supplementing VM diets with MET sources in their purified forms is a prerequisite to keep fish growth performances and health [2,3].

Nowadays, different sources of MET are used in animal nutrition with DL-MET and DL-2-Hydroxy-4-(methylthio) butyric acid (a structural analog of MET that can be converted into MET and hereafter referred to MHA for Methionine Hydroxy Analog), being the most widely used MET sources in the field. Nonetheless, and beyond the fact that both sources can be used as a MET source, knowledge about fish performances are scarce and still debated when directly comparing their efficiencies. For instance, while studies performed in rainbow trout [4], sunshine bass [5], common carp [6], Nile tilapia [7] and channel catfish [8]) showed that DL-Met is a better MET source than MHA with respect to their growth performances, other studies (e.g., in turbot [9] or channel catfish [10]) concluded that MHA is used as efficiently or even better than L-MET or DL-MET. Many hypotheses could explain these opposite conclusions, such as differences in diet compositions, concentrations used in relation to the Met requirement of the species and product forms used, as well as the species studied, but none of these studies investigated the molecular mechanisms that drive the effectiveness of a said supplementation compared to another. At the molecular level, one possible explanation would be that both molecules are not uptaken by the same transporters. MHA absorption likely occurs via monocarboxylate transporters (MCTs) rather than canonical AA transporters, as proposed in other species [11] but also in RT gut tissues [12]. Such differences in MET source absorptions due to fish species or MET forms might impair the supply of MET-related pathways, which control part of anabolic and catabolic processes responsible for the healthy growth of organisms. The ability to answer this question will certainly help to better adapt fish diet formulations according to the metabolic properties of farmed fish species considered.

In the present study, we took advantage of the in vitro approach and used RT cell lines to decipher molecular mechanisms related to fish nutrition [13]. The use of a nutritional in vitro approach in which the growing media of cell lines are only and fully depleted for MET-related metabolites (e.g., MET and cysteine—CYS) and supplemented back with different concentrations of MET or MHA allowed us to discriminate any differences in the efficiencies of both synthetic forms to supply the cellular metabolic pathways related to MET. Among these pathways, three received particular attention, namely the MET-related transmethylation and trans-sulfuration pathway, the Integrated Stress Response (ISR) pathway and the mechanistic Target Of Rapamycin (mTOR) pathway. The first mentioned consists of the transformation of MET in a series of reactions in secondary metabolites with multiple functions such as S-Adenosyl Methionine (SAM, an important methyl group donor notably involved in epigenetic regulations), CYS (a semi-essential proteinogenic sulfur-containing amino acid) and glutathione (GSH; the most abundant thiol in humans carrying antioxidant functions and being the major cellular redox buffer). Directly linked to this pathway is the mTOR pathway. mTOR is a serine threonine kinase that forms two protein complexes, mTORC1 and mTORC2. mTORC1 is in charge of sensing nutrient availability such as AAs [14], prior to being activated and phosphorylating different protein targets [15] (e.g., S6 kinase—S6K and Eukaryotic translation initiation factor 4E-binding protein 1—4EBP1). Once phosphorylated, these targets allow anabolism to take place by stimulating protein, lipid and carbohydrate metabolisms to finally promote cell growth and proliferation. Notably, not all AAs are sensed by the mTOR amino acid sensing machinery. Only the following four were clearly established as signaling molecules: leucine (LEU), arginine (ARG), glutamine (GLN) and MET [16]. Interestingly, the MET-induced mTORC1 activation does not rely on MET itself but on one of its secondary metabolite, SAM [17], linking directly the MET-related metabolic pathway activity to the mTOR pathway. Finally, and unlike the two pathways mentioned previously, the integrated stress response (ISR) pathway [18] is activated following AA deprivation through various interconnected axes, not yet fully described. Among these is the unfolded protein response (UPR), which is driven by the upregulation of the *X-box binding protein 1* (*XBP1*) *gene*, and the General Control Nonderepressible 2 (GCN2) pathway, which is activated via the interaction of uncharged tRNA with GCN2 kinase in charge to repress general protein synthesis and stimulates the expression of AA biosynthetic genes such as *Asparagine Synthetase* (*ASNS*). These pathways have well-described mechanisms, which are required to cope with AA restriction conditions. Interestingly, when AA deprivation conditions persist or are too extreme, the GCN2 pathway could also be responsible for the induction of cell death program type I through the strong overexpression of DNA damage-inducible transcript 3 (DDIT3, also known as the C/EBP homologous protein—CHOP) described as a proapoptotic transcription factor [19]. 

Since differences in DL-MET and MHA absorption might differentially fulfill RT metabolic needs, and because liver is one of the most metabolically active organs fueled by newly uptaken AAs, we decided to perform our experiments using two RT liver-derived cell lines called RTH-149 [20] and RTL-W1 [21] from the available RT Invitromatic [22]. Thus, based on the knowledge gathered and protocols set up from our previous study [13], combined with the use of cutting-edge technologies (e.g., UPLC-Mass Spectrometry), we conducted a full set of experiments, from cell proliferation to MET-related intracellular metabolites analysis, to compare the efficiencies of DL-MET and MHA and to regulate the aforementioned three MET-related pathways governing cellular homeostasis. 

## 2. Results

### 2.1. Only DL-MET Supports Cell Proliferation

**Figure 1:**
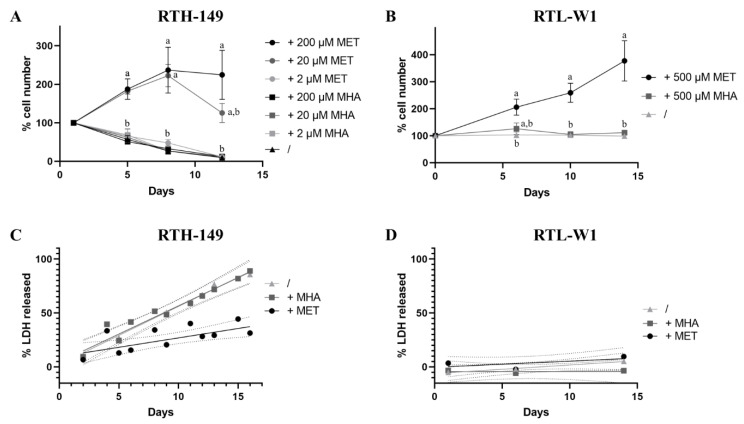
Differences of the effect of MET sources on cell proliferation and cell death rates. (**A**,**B**) Cell proliferation assays were performed with MET-depleted media (/) or supplemented with indicated concentrations (2-, 20-, 200- or 500 µM) of MET and MHA. Cells were counted at various time points to establish the proliferation curve for RTH-149 cells (**A**) and RTL-W1 (**B**). Results from 3 independent experiments are presented as the mean +/− SEM for each time point. Conditions showing results statistically different from each other are presented using a different letter (one-way ANOVA with Tukey’s post hoc test). (**C**,**D**) Cytotoxicity assays were performed with MET-depleted medium (/) alone or supplemented with 200 µM or 500 µM of MET or MHA for RTH-149 (**C**) and RTL-W1 cells (**D**), respectively. Results from 3 independent experiments are shown as the measured LDH-released means of triplicates as ● (+MET), ■ (+MHA) or ▲ (/) for each time point assessed and solid lines representing the calculated linear regressions for each treatment. Only calculated R-squared for RTH-149 cells untreated (/) or supplemented with MHA (+MHA) showed a correlation (R² > 0.5) between treatment and the % of LDH released over time with R² = 0.918 and 0.878, respectively. Dashed lines represent the calculated 95% confidence intervals (CI) of the corresponding conditions where nonoverlapping CI are considered statistically different at a given time point.

As mentioned before, debates still exist in the fish nutrition field to determine if synthetic MET forms are equivalent to promote fish growth when supplemented for MET-deficient diets. Therefore, to address this question, cell proliferation assays were initially performed with RTH-149 and RTL-W1 cells grown in media recapitulating the complete formulation of their regular growing media (respectively, MEM and L-15 media), with the exception that each medium was depleted for MET and CYS. The choice to also deplete the media for CYS was mainly motivated by the fact that media supplementation with CYS, described in most cell lines as a nonessential AA, would have to supply part of the MET–metabolic pathway and therefore might have hidden some of the phenotypes caused by MET deprivation. Thus, by supplementing or not these media with DL-MET (+MET) or MHA (+MHA) at different concentrations, and by counting adherent cells at various time points over 12 days, the RTH-149 cell proliferation curve (Figure 1A) clearly demonstrates that, whatever the concentration of MHA added to the growing medium, the cells display a strong growth arrest comparable to the one observed for cells grown in the absence of supplementation.

However, while 2 µM of DL-MET supplementation did not allow to observe cell proliferation, when added at 20 or 200 µM, corresponding, respectively, to 10 and 100% of the methionine concentrations normally used in their regular media, DL-MET clearly allows the proliferation of cells that reached 100% confluence in about 8 days. Nonetheless, it seems that 20 µM of DL-MET supplementation is rapidly consumed by cells and are limited from 8 days of growth. In the meantime, similar experiments were conducted in RTL-W1 cell lines, during which we discovered that CYS deprivation causes cell death induction with either of the two supplemented MET sources (data not shown). Since RTL-W1 cells displayed a strong dependency on CYS availability, all the experiments performed with this cell line had to be conducted in CYS-containing media. In alignment with the results obtained in RTH-149 cells, RTL-W1 cell proliferation could only be observed upon DL-MET supplementation but not MHA (Figure 1B). 

Aware that MET deprivation can induce cytotoxic stresses leading to cell death, we sought to investigate if differences in cell death rates can explain the differences in cell proliferation observed when cells were grown in the presence or absence of the different MET forms (Figure 1C,D). Indeed, cytotoxic assays performed in RTH-149 cells showed a similar and strong induction of cell death upon either MET deprivation or MHA supplementation conditions, while, when DL-MET was added to the growing media, the cell death rate stayed at more basal values. Surprisingly, when similar experiments were conducted in RTL-W1 cells, no statistical differences were observed between the three different nutritive conditions, demonstrating that the differences in cell proliferation previously observed cannot be explained by excessive cell death inductions in this cell line. 

Altogether, these first results clearly demonstrated that, in both RTH-149 and RTL-W1 cell lines, the two MET forms are not equivalent when considering the cell proliferation and cell death phenotypes. Therefore, further investigations were conducted to understand which pathways could be differently impaired when MHA supplementation was used in comparison to DL-MET. 

### 2.2. Responses of Cellular AA Sensing Pathways to Different MET Sources

Since strong differences were observed in the proliferative capacities of cells grown in presence of various MET forms; two distinct pathways were then assessed for their responses in RTH-149 and RTL-W1 cells, namely the mTOR and ISR pathways. As described before in RTH-149 cells, these two pathways are known to be regulated by AA availability but in opposite ways. Indeed, when the mTOR pathway is activated by the presence of AA, the ISR is supposed to be kept inactive, while AA deprivation leads to mTOR inactivation and switches on the ISR through multiple axis involving, but not restricted to, GCN2 activation. Thus, the growth of cells in the presence of media containing or not different MET forms allowed us to evaluate the activation status of both pathways by means of Western blot and qPCR analysis.

**Figure 2:**
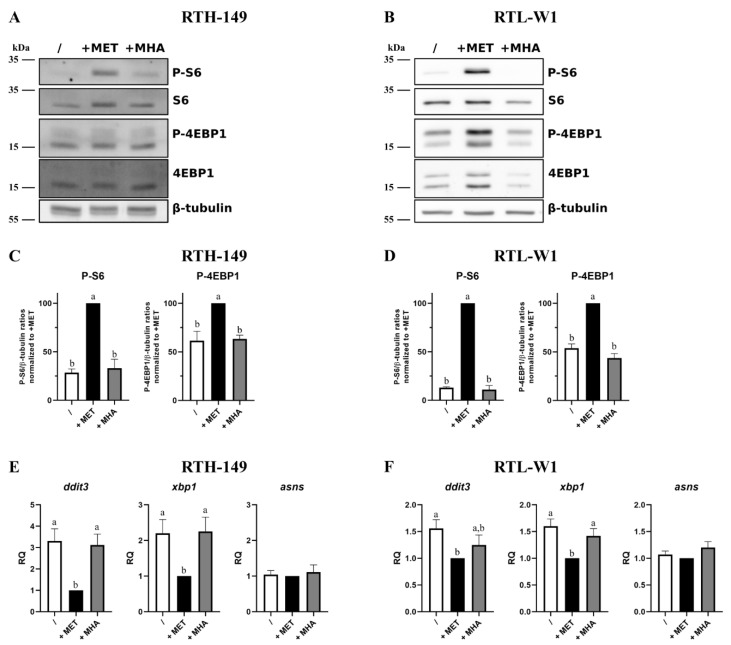
Impact of MET sources on mTOR and ISR pathways. (**A**,**B**) Representative images of phosphorylation levels of mTOR targets: 4EBP1 and S6, assessed by Western blot following 5-h treatments with MET-depleted media (/) supplemented with 200 µM or 500 µM (for RTH-149 cells (**A**) or RTL-W1 cells (**B**), respectively) of MET or MHA. (**C**,**D**) Densitometry analysis of the phosphorylation levels of mTOR targets in RTH-149 (**C**) and RTL-W1 (**D**) of 5 independent experiments. Data represent the Phospho targets levels in β-tubulin ratios normalized to +MET conditions. Conditions showing results statistically different from each other are presented using a different letter (one-way ANOVA with Tukey’s post hoc test). (**E**,**F**) ISR pathway activation quantified via gene expression analysis by RT-qPCR of *ddit3*, *asns* and *xbp1* following 24-h (**E**) or 16-h (**F**) treatments with MET-depleted media (/) supplemented with MET (+MET) or MHA (+MHA) for RTH-149 cells (**E**) (*n* = 7) or RTL-W1 cells (**F**) (*n* = 5) using similar concentrations as described in (**A**,**B**). Conditions showing results statistically different from each other are presented using a different letter (one-way ANOVA with Tukey’s post hoc test).

The results presented in Figure 2A,B clearly showed strong phosphorylation levels of two, direct and indirect, mTOR targets (namely 4EBP1 and S6 proteins, respectively) only when cells were grown in DL-MET-containing media. Densitometry analysis of independent experiments (Figure 2C,D) confirmed these observations and revealed that only DL-MET-supplemented media, whatever the cell line considered, significantly increased the mTOR activation levels, while MET deprivation and MHA supplementation conditions displayed minor mTOR activation levels. For these two last conditions, the presence of a basal activation level, even low, could be caused by other amino acids contained in the media such as leucine (LEU), arginine (ARG) and glutamine (GLN). On the other hand, the activation levels of the ISR pathway were assessed in the RTH-149 and RTL-W1 cell lines (Figure 2E,F, respectively) by means of RT-qPCR analysis by measuring the expression levels of some ISR-specific genes already established as molecular markers of ISR activation in fish cell lines [13]. Clearly, the results show the exact same trend of *ddit3*, *xbp1* and *asns* gene expression profiles in both cell lines with respect to the growing conditions considered. Indeed, MET deprivation leads to the induction of *ddit3* and *xbp1* expressions in RTH-149 cells and, to a lesser extent but still significant, in RTL-W1 cells. Interestingly, *asns* gene expressions were kept constant at the basal levels whatever the growing condition considered. Furthermore, in line with our previous experiments, MHA supplementation did not manage to restore the basal expression levels of *ddit3* and *xbp1* genes, indicating that the ISR pathway was still activated despite the addition of this MET form in the growing media. 

Altogether, and beyond providing new insights on molecular pathways that are differentially activated by MET forms, these results further supported the proliferative phenotypes shown in Figure 1, since (1) mTOR is described as a central hub that governs anabolism and, therefore, cell growth and proliferation, and (2) the ISR pathway is known to repress general protein synthesis and, in specific cases, to orchestrate an apoptotic response through DDIT3 upregulation. Therefore, further efforts were engaged to understand if MHA displayed differences in its ability to be metabolize when compared to DL-MET, which could explain, at least in part, if not all, the phenotypes observed so far.

### 2.3. Assessing the MET-Related Metabolic Pathways When It Is Fueled with DL-MET or MHA

**Figure 3:**
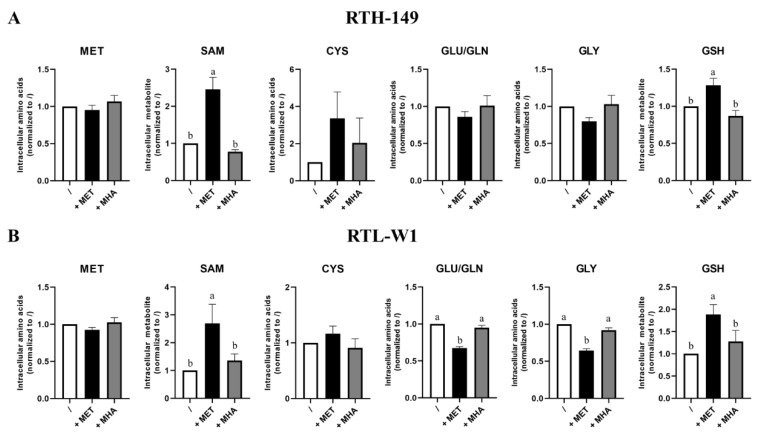
MET sources impact on the intracellular level of MET-related metabolites. The intracellular levels of SAM and GSH were determined by HPLC-UV and intracellular levels of MET, GLY, GLU/GLN and CYS by HPLC-FL following 24 h of treatment with MET-depleted media (/) supplemented with 200-µM or 500-µM (for RTH-149 cells ((**A**), *n* = 6) or RTL-W1 cells ((**B**), *n* = 8), respectively) of MET (+MET) or MHA (+MHA). Conditions showing results statistically different from each other are presented using a different letter (one-way ANOVA with Tukey’s post hoc test).

Since both RTH-149 and RTL-W1 cell lines showed proliferative phenotypes and expected responses for the mTOR and ISR pathways when grown in the presence of DL-MET but not MHA, we first examined the transcriptional regulations of the genes involved in the MET-related metabolism pathways (Supplementary Figure S1). First of all, we noticed that, among all the genes tested, nine showed a specific regulation by MET forms availabilities in at least one of the two cell lines considered. Interestingly, two of these genes, namely *mat2aa* and *gclc*, showed consistent regulations in RTH-149 and RTL-W1 cells according to DL-MET availabilities in the growing media. Indeed, MET deficiencies, together with MHA supplementations conditions, led to an upregulation of *mat2aa* gene expression, while the *gclc* gene appeared upregulated only in the presence of DL-MET in both cell lines. Besides having characterized for the first time these cell lines for the expression of these enzymes, it is noteworthy to mention that, again, the transcriptional regulations observed upon DL-MET or MHA supplementation corroborated our previous findings demonstrating that DL-MET and MHA are not biologically equivalent in the fish cell lines tested. Hence, intracellular analyses of MET-related metabolites have been conducted and assessed in more integrated ways (Figure 3).

Astonishingly, the results showed no difference of intracellular MET and CYS contents, whatever the growing condition, considered in both cell lines, not even in DL-MET-treated cells (Figure 3). However, significant evidence of MET metabolization occurred when measuring intracellular SAM contents, as well as glutathione (GSH, a tripeptide that can be considered as the end product of the trans-sulfuration pathway). Again, only DL-MET treatments led to a significant increase of intracellular SAM and GSH, while MHA supplementation showed similar levels of metabolites for those measured upon MET deprivation conditions. Furthermore, we noticed that the glutamine/glutamate (Gln/Glu) intracellular pool, as well as glycine (Gly), showed similar profiles in RTH-149 and RTL-W1 cells, with a decrease in DL-MET-treated cells. Knowing that Glu and Gly are two amino acids required for GSH synthesis, these decreases could therefore reflect the consumption necessary for the increase of GSH synthesis, together with the protein synthesis needed to support cell proliferation observed only in DL-MET treatments. Moreover, it is important to notice that intracellular SAM contents perfectly corroborated the mTOR activation levels measured in both cell lines (Figure 2C,D), further supporting the conclusions for which only DL-MET supplementation allows mTOR activation and its subsequent outcomes in cell proliferation. 

Finally, since all the experiments conducted so far lead to the same conclusion that MHA supplementation is biologically ineffective for RTH-149 and RTL-W1 cells, the reasons for this defect in metabolic functions had to be understood at the molecular level. 

### 2.4. A Defect in MHA Uptake and Metabolization?

**Figure 4:**
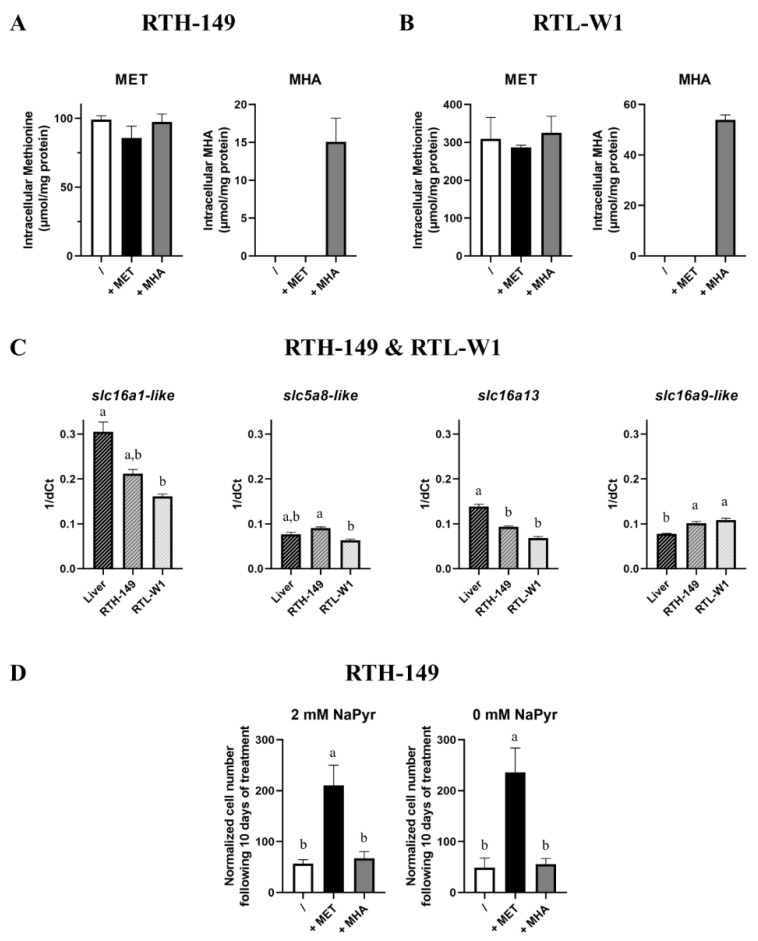
MET sources uptake. (**A**,**B**) Intracellular level of MET and MHA were determined by UPLC-MS following 24 h of treatment of cells with MET-depleted media (/) supplemented with 200 µM or 500 µM (for RTH-149 cells ((**A**), *n* = 3) or RTL-W1 cells ((**B**), *n* = 3), respectively) of MET (+MET) or MHA (+MHA). (**C**) Monocarboxylate transporters expression (slc5a8-like, slc16a13, slc16a9-like and slc16a1-like assessed by RT-qPCR for both RTH-149 (*n* = 3) and RTL-W1 (*n* = 3) cell lines compared to in liver tissues (*n* = 9). (**D**) Proliferation assay of RTH-149 cells 10 days following treatment with MET-depleted media (/) supplemented with 200 µM of MET (+MET) or MHA (+MHA) in the absence or presence of 2-mM sodium pyruvate (*n* = 3). Conditions showing results statistically different from each other are presented using a different letter (one-way ANOVA with Tukey’s post hoc test).

Since only L-MET can be directly used by cells, and because no increase in intracellular MET content (or MET-related metabolites content) was observed upon MHA supplementation compared to MET deprivation conditions, it appeared likely that the cell lines could not express the set of enzymes required to convert MHA (a racemic mixture of D-MHA and L-MHA) into L-MET. Indeed, it is known that DL-MHA (as well as D-MET) needs to be first oxidized in 2-keto-(4-methylthio)butanoic acid (KMB) prior to being converted in L-MET following a transamination reaction [23]. If the second reaction can be ensured by multiple transaminases, the specific oxidations of L-MHA, D-MHA or D-MET are catalyzed by stereospecific enzymes encoded by *hydroxyacid oxidase* 1 (*hao*1), *lactate dehydrogenase d* (*ldhd*) and *d-amino acid oxidase* (*dao*) genes, respectively. Therefore, the expression of these genes was measured and compared to the expression observed in RT liver tissues (Supplementary Figure S2). The analysis revealed that, even though a lower expression of *hao1* was observed in both cell lines when compared to liver tissues, all the three enzymes were found expressed in both cell lines. It therefore appeared unlikely that the cellular proliferation and signaling defects were only caused by a decrease in L-MHA isoform conversion, since the cells could use D-MHA to fuel their metabolism. Therefore, the possibility of an absorption defect was carefully explored (Figure 4).

First of all, because MHA carries a hydroxyl group instead of the α-amino group, it was therefore impossible to measure the intracellular MHA contents by means of the derivatization and fluorescent detection of the amino acid technique commonly used in analytical chemistry. Thus, following the implementation of a specific protocol for the simultaneous detection of MET and MHA via a mass spectrometry analysis, experiments were conducted to measure the absolute content of MET and MHA at the stationary state in both cell lines grown in MET-deprived media containing or not DL-MET or MHA (Figure 4A,B). First of all, in alignment with the previous results obtained via another detection mode, no statistical differences between the three growing conditions were measured for the intracellular MET contents in both cell lines. However, we noticed that, despite being absorbed by RTH-149 and RTL-W1 cells, MHA concentrations ranging from 15 to 50 µmol/mg of protein in RTH-149 and RTL-W1 cells, respectively, were five to six times lower than the MET concentrations (~90 µmol/mg of protein in RTH-149 cells and 300 µmol/mg of protein in RTL-W1 cells). Therefore, one likely hypothesis was that RTH-149 and RTL-W1 cells were not expressing the monocarboxylate transporters (MCTs) proposed to be in charge of MHA import. Surprisingly, and as shown in Figure 4C, both cell lines expressed the MCTs in quantities close to those measured in RT liver tissues, with only slight but statistical differences (positively and negatively) in their expression levels. Finally, since MHA carries chemical properties that are similar with pyruvate (a compound usually supplemented in cell culture media to support high metabolic needs and cell proliferation rates of cell lines), we wondered if pyruvate contained in growing media would compete for MHA uptake, as pyruvate is a known substrate of MCTs. Thus, RTH-149 cell proliferation assays (Figure 4D), performed in media containing or not 2 mM of sodium pyruvate (NaPyr), indicated that (1) cells did not rely on pyruvate availability, since similar proliferation rates were observed in the absence and presence of pyruvate and (2) the lack of pyruvate did not even improve the proliferation of RTH-149 cells grown with MHA as the MET source, indicating that pyruvate did not compete for MHA uptake in cells. 

## 3. Discussion

For the past decades, the incremental use of plant ingredients in aquafeed formulations (nowadays, about 80% of regular commercial diets are constituted of plant proteins) allowed to sustain farmed fish production without affecting fish growth performances. Nonetheless, efforts should be pursued to totally replace FM with VM to meet the United Nation’s expectations by 2030, notably with respect to the Sustainable and Development Goal 14 (SGD 14), which directly relates to fisheries and aquaculture [24]. Alongside the increase of plant proteins in fish diets is the increase in supplementation of these diets with synthetic amino acids, since plant proteins display unbalanced amino acid compositions, especially for MET (the most restricted EAA in plant proteins compared to animal proteins). Thus, in proportion, MET supplementation will further increase in fish diets. Here, the question of the most appropriate supplemental MET forms to use is still open, especially when comparing DL-MET and MHA efficiencies. For many other animals like mammals, birds and, also, specific fish, the equimolar bioefficacy of MHA in comparison to DL-MET has been shown to be only around 74%. These differences in bioavailability between these two forms were previously established, with differences in intestinal fluxes recently proposed [6,7] as a likely explanation; however, questions still remain to be elucidated with respect to the outcomes of these differences in other tissues. To address this question, RT liver-derived cell lines were used, as a simplified model, to extrapolate the unique and specific effects of DL-MET or MHA on RT liver functions. Indeed, the absence of post-prandial cross-talks that could exist between organs, and which affect the transcriptional landscape of remote organs, allows to reveal intrinsic phenotypes related to the tissue when exposed to the different MET sources. As a result, and following a full set of experimentations, the conclusions drawn from this study clearly highlighted a strong defect in MHA absorption in RTH-149 and RTL-W1 cells when compared to DL-MET. As a consequence, MET-related metabolic pathways are not sufficiently supplied, exemplified by the absence of an increase of SAM and GSH synthesis upon MHA treatments and when compared to DL-MET treatments. Therefore, opposite signaling pathways are activated whether MHA or DL-MET are used as synthetic MET sources. Indeed, while DL-MET, through SAM-dependent mTOR activation, allows anabolic signaling that drives cell proliferation, MHA induces activation of the catabolic pathway, notably through ISR activation, leading, at least, to cell growth arrest or even cell death, depending on the cell line. Altogether, these results shed new light on the differences that could exist in livers when MHA and DL-MET are used as supplemental MET sources in MET-restricted fish diets.

Moreover, important information was gathered on the biology of the two fish cell lines studied, contributing to their further characterization required to finetune our understanding of the mechanisms related to fish nutrition recently initiated [13]. For instance, and beyond the discovery of the cysteine dependency of the RTL-W1 cell line, we could observed that, in both cell lines, *asns*, a GCN2 target gene previously described to be upregulated following total AA starvation [13], did not appear to be regulated in a similar manner upon MET starvation conditions, since no upregulation were observed. One explanation would be that GCN2 activation could be dampened because of the presence of all the other AAs in the growing media when compared to the total AA starvation response. This hypothesis is further supported by the expression levels of *ddit3* measured in RTH-149 cells, since only a three-fold change was quantified, while, as previously measured [13], the total AA starvation led to an upregulation twice as important (six-fold change). Therefore, considering that total AA starvation induced *asns* upregulation by only three-fold change [13], a milder GCN2 activation induced by unique MET restriction could limit *asns* upregulations, which would therefore fall below the technical and biological detection levels. Nonetheless, further investigations using cell lines could help to define, if existing, the gradual response of the GCN2 pathway by correlating the upregulation levels of a list of described targeted genes with various AA restriction conditions (qualitatively and quantitatively). Such results would offer new markers to discriminate in vivo the most suitable diet formulations with respect for their AA compositions and sources. 

Another observation made from this study is that, in light of the analysis performed to quantify intracellular MET-related metabolites, MET seemed to be quickly and constantly metabolized in cells. Indeed, none of the analyses performed in this study allowed to detect significant variations of its concentrations, whether or not it was available in the extracellular environment. MET is certainly one of the AA showing the lowest plasma concentration in RT [25] but, also, in humans [26]. Therefore, we can speculate that, similar to how glucose is rapidly phosphorylated into glucose-6 phosphate to avoid its transport back to the extracellular environment [27], MET is also rapidly metabolized, keeping its concentration at constant and basal levels in cells. Following this hypothesis, it would make sense that the mTOR AA-sensing machinery is capable to detect SAM but not MET [17]. Indeed, since mTOR is a central hub in charge of integrating environmental signals, among which is AA sufficiency, to orchestrate metabolism and to promote cell growth and proliferation, SAM is therefore the best signaling molecule correlated with extracellular MET availability. As a consequence, it appears crucial in the future to evaluate the suitability of said diet by measuring MET plasma concentrations in fish together with SAM intracellular contents in various tissues to establish if MET requirements are well-covered in the whole organism, notably in muscle, the most growing tissue in RT. 

Finally, the analysis performed to measure the intracellular MHA content brought fundamental and technical questions related to MHA and, notably, to its uptake. Indeed, we could observe that the MHA intracellular content is low. Of course, this concentration certainly does not reflect the net amount of MHA that is uptaken by cells, since it is described that MHA is rapidly converted into MET once absorbed [11]. Nonetheless, the whole set of experiments led us to conclude that MET-related metabolic pathways were not sufficiently fueled by MHA. Indeed, no difference in secondary metabolites were observed in between cells deprived of MET or supplemented with MHA, while DL-MET supplementation allowed a marked increase in SAM and GSH intracellular contents. This also disproved the hypothesis that some intrinsic defects of this pathway would be carried by both cell lines. Likewise, defects in the ability of cells to convert MHA into KMB were excluded, since an accumulation of MHA should therefore be observed, unlike the small amount detected in cells. It is also noteworthy to mention that the functional invalidation of all the cellular transaminases that can convert KMB into L-MET [23] is very unlikely to have occurred during the immortalization processes of both cell lines without inducing the loss of these cell lines. Finally, the most conceivable explanation could be that MHA is not efficiently uptaken by these cells. Nevertheless, when the expression of MCTs proposed to be in charge of intestinal MHA flux [11,12] were assessed in the two hepatic cell lines, all of them were detected at mRNA levels. To date, it cannot be excluded that post-transcriptional/translational regulations, together with post-translational modifications, are critical for MCTs activities. In this line, future research developing molecular tools that currently miss in farmed-fish related studies (e.g., gene invalidation and fish-specific antibodies) will considerably help to move forward our understanding of the biology of fish. 

To conclude, this study offered new insights in term of molecular mechanisms responsible for the defect of MHA absorption shown in RTH-149 and RTL-W1 cell lines. However, further investigation is still required to fully understand the differences of the MET sources utilized by other cell types or cell lines. Future research using gut cell lines [28,29] could be of great interest for this purpose, notably by comparing their MHA contents and metabolization rates to those obtained in the present study. Moreover, a feeding trial comparing DL-MET and MHA efficiencies could also be very insightful, notably if tracking specifically MHA in the main tissues of the organism. Indeed, by extrapolating the results obtained from this study to liver functions, it seems that livers would only rely on MET provided by the digestion of dietary proteins. Moreover, to the best of our knowledge, no study has yet conducted a muscle analysis of trout fed a MHA-supplemented diet. The ability of fish muscles to uptake and metabolize MHA to support the constant growth of myocytes is also a very important question to address if we want to help fish farmers choose the most appropriate synthetic MET form for their fish productions. Hopefully, future advances in the establishment of new cell lines (e.g., RT myocyte cell lines are still deeply missing in the field) will considerably help to move forward our understanding of the biology of fish of agronomic interest.

## 4. Materials and Methods

### 4.1. Cell Culture and Treatments

Two cell lines derived from rainbow trout were used. The RTH-149 hepatoma cells (ATCC^®^ CRL-1710, LGC Standards, Molsheim, France) were routinely grown in minimum essential medium (MEM, #61100, Thermo Fisher scientific, Waltham, MA, USA) supplemented with 10% nonessential amino acid (NEAA) solution (#11140-50), 10% fetal bovine serum (FBS, #10270-106), 2-mM sodium pyruvate (NaPyr, #11360-070), 100-units/mL penicillin and 100-g/mL streptomycin (#14065-056), all provided by Gibco (Thermo Fisher scientific) and 25-mM HEPES (#BP299-1, Fisher Bioreagents, Fisher Scientific SAS, Illkirch Graffenstaden, France). For the RTL-W1 cell line, cells were grown in Leibovitz’s L-15 medium containing 10% FBS, 100-units/mL penicillin and 100-g/mL streptomycin and 25-mM HEPES. For both cell lines, cells were maintained at 18 °C, the medium was replaced twice a week and cells were passaged at 80% of confluence. Cell counting was achieved using a Cellometer K2 (Nexcelom Bioscience LLC, Lawrence, MA, USA) to plate cells prior to the experiments. Cells were seeded at a density of 50–60% for RNA isolation and 40–50% for protein extraction in 5-cm dishes, a density of 20% in 12-well plates for proliferation and cytotoxicity assays and a density of 80–90% in 6-well plates for liquid chromatography experiments. Cells were incubated at 18 °C over 2 days prior the treatments and were washed twice with PBS before the exposure to the appropriate treatment.

For the RTH-149 cell line experiment, Dulbecco’s modified Eagle’s medium (DMEM) without MET and CYS was used (C4030, Genaxxon Bioscience, Ulm, Germany). The medium was supplemented with 1-g/L glucose, 25-mM HEPES, 2-mM L-glutamine (#61100-053, Gibco, Thermo Fisher Scientific, Waltham, MA, USA), 2-mM NaPyr, 4-nM insulin (#10516, Sigma-Aldrich, Darmstadt, Germany) and 10% FBS. This MET-depleted medium (/) was supplemented or not with 2, 20 or 200 µM of DL-MET (+MET) (Evonik, Essen, Germany) or 2, 20 or 200 µM of MHA (+MHA) (#55875, Sigma-Aldrich). For RTL-W1 cells, Leibovitz’s L-15 medium without MET and CYS was used (C4063, Genaxxon Bioscience) supplemented with 8.5-mM NaCl, 1-mM CYS and 10% FBS. This medium (referred to as “/”) was supplemented or not with 500 µM of DL-MET (+MET) (Evonik) or 500 µM of MHA (+MHA) (#55875, Sigma-Aldrich). For all RT-qPCR analyses, cells were treated with the MET-depleted medium supplemented or not with DL-MET and MHA during 24 h for RTH-149 cells and 16 h for RTL-W1. For both cell lines, WB analyses have been conducted following 5 h of treatment with media described above but in the absence of insulin and FBS to avoid amino acid-independent mTORC1 activation.

### 4.2. Proliferation Assay

Two days prior the treatment, cells were plated in their regular media, which was renewed the following day. Cells were treated with MET-depleted media described previously and were counted at different time points, indicating that Cellometer K2 with the AO/PI reagent (Nexcelom) was used according to the manufacturer’s instructions. For each time point, a number of 3 wells/condition were counted and normalized to the first time point of the experiment. Results showed a representative experiment from 3 independent experiments ± SD.

### 4.3. Cytotoxicity Assay

Two days prior the treatment, cells were plated in the appropriate medium that was renewed the following day. Cells were treated and cytotoxicity was measured using the CyQUANT LDH Cytotoxicity Assay kit (#C20300, Thermo Fisher Scientific) following the manufacturer’s instructions. The results shown were generated from 3 independent experiments performed for each cell line.

### 4.4. Protein Extraction and Western Blot Analysis

Following a cell wash with ice-cold PBS, cells were lysed using RIPA buffer (#89901, Thermo Scientific) containing a Halt protease and phosphatase inhibitor cocktail (#78442, Thermo Scientific). After 30 min of incubation on ice, samples were centrifuged at 12,000× *g* at 4 °C for 10 min. The supernatant was collected, and the protein concentrations were measured using the bicinchoninic acid method (#BCA1-1KT, Sigma-Aldrich). Equal amounts of protein were mixed with Laemmli buffer prior to being subjected to 12% sodium dodecyl sulfate polyacrylamide gel electrophoresis (SDS-PAGE) for protein separation. Then, proteins were transferred onto polyvinylidene fluoride (PVDF) membranes (#IPFL00010, Merk Millipore, Burlington, MA, USA). The membranes were incubated with the following primary antibodies: anti-ribosomal protein S6 (#2217; Cell Signaling Technologies, Danvers, MA, USA), anti-phospho-S6 (Ser235/Ser236, #4856; Cell Signaling Technologies), anti-4EBP1 (#9452; Cell Signaling Technologies), anti-phospho-4EBP1 (Thr37/Thr46, #9459; Cell Signaling Technologies) and anti-β-tubulin (#2146; Cell Signaling Technologies). Following several washes, membranes were incubated with secondary antibodies. For samples from RTH-149, IRDye secondary antibody was used (#926-68071, LI-COR, Inc., Lincoln, NE, USA), and signal acquisition was performed by infrared fluorescence with the Odyssey^®^ Imaging System (LI-COR, Inc.). For samples from RTL-W1, membranes were exposed to HRP-labeled goat anti-rabbit IgG secondary antibody (#31460, Thermo Fisher scientific) and incubated in SuperSignal™ West Pico PLUS Chemiluminescent Substrate (#34580, Thermo Fisher Scientific). Chemiluminescence acquisition was performed with the iBright 1500 imager (Thermo Fisher Scientific). For both cell lines, quantification of the protein expression was performed using ImageJ software (NIH, Bethesda, MD, USA), using β-tubulin as a loading control for phosphorylation signal normalizations. 

### 4.5. RNA Extraction and RT-qPCR Analyses

For both cell lines, cells were washed with PBS prior to RNA extraction and purification using the RNeasy mini kit (#74104, Qiagen, Hilden, Germany) following the manufacturer’s instructions. RNA concentration and purity were evaluated using a Nanodrop^®^ ND 1000 spectrophotometer (Marshall Scientific, Hampton, NY, USA) and stored at −80 °C. The cDNA for the gene expression analysis was synthetized from 1 µg of RNA from RTH-149 cells and 500 ng of RNA from RTL-W1 using the Superscript III RNAseH reverse transcriptase kit (#18080-093, Invitrogen, Carlsbad, CA, USA) with random hexamers (#C1101, Promega, Madison, WI, USA). For all genes analyzed, the reverse transcription (RT) reactions were carried out following the manufacturer’s instructions in a thermocycler. After the first step of denaturation (65 °C; 5 min), the run followed the conditions: 25 °C for 5 min, 55 °C for 1 h and 70 °C for 15 min, then held at 4 °C. The real-time quantitative PCR reactions were performed in triplicate. For the reaction, 3 µL of Light Cycler^®^ 480 SYBR Green I Master (Roche, Bâle, Switzerland) were used for 2 µL of 1/40 diluted cDNA with 0.24 µL of each gene-specific primer (at 10 µM) (listed in Table 1) and 0.52 µL of nuclease-free water. The reaction was carried out with a Roche Light Cycler 480 system (Roche). The RT-qPCR reaction was initiated at 95 °C for 10 min, followed by 45 cycles of a 3-step amplification program (95 °C for 15 s, 60 °C for 10 s and 72 °C for 15 s). At the end of the reactions, melting curves were monitored to confirm the specificity of the amplification reaction. Negative controls (RT- and cDNA-free samples) were systematically included to each run. Since ef1α was found to be the most constant and accurate housekeeping gene tested, it was used for the normalization. All the gene expression results were presented as the relative quotient (RQ) calculated using the ∆∆Ct method or 1/∆Ct when comparing gene expressions from the cell lines with RT liver tissues. No fish had to be sacrificed for the purpose of this study, since the RT liver samples were obtained from a previous experiment [30].

### 4.6. Liquid Chromatography Procedures

#### 4.6.1. Methanolic Extraction of Polar Metabolites

For both cell lines, after 24 h of treatment, cells were washed 3 times with ice-cold PBS. Polar metabolites were extracted using a mix of MeOH/H2O (8/2, *v*/*v*) and incubated 5 min on a rotary shaker at 4 °C followed by a centrifugation at 16,000× *g* during 10 min at 4 °C. Supernatants were transferred in appropriates liquid chromatography vials.

#### 4.6.2. SAM and GSH Separation and Detection by HPLC-UV

Intracellular S-adenosyl-L-methionine (SAM) and reduced glutathione (GSH) levels were measured using a Waters^®^ Alliance System (2695 separation module) equipped with a Waters^®^ 2695 Alliance autosampler and supported with a Waters^®^ 2487 dual λ Absorbance Detector (Waters, Milford, MA, USA). Chromatographic separation was achieved on a SymmetryShield RP18 column (4.6 mm × 150 mm, i.d. 3.5 μm). The column was operated at 30 °C. The injection volume was 50 μL, and the flow rate was set at 0.7 mL/min. A ternary solvent system was used consisting of (A) pH 2.70 ± 0.05 20-mM phosphate buffer, (B) methanol and (C) acetonitrile. The mobile phase was filtered through in-line 0.2-μm membrane filters. The following gradient elution was employed: 0–10 min: 99.5% A, 0.5% B; 12 min: 40% A, 60% C; 12–15 min: 40% A, 60% C; 17 min: 99.5% A, 0.5% B and 17–30 min (column equilibration): 99.5% A, 0.5% B. The eluate was monitored with a double wavelength UV detection: 210 nm for GSH and 258 nm for SAM.

#### 4.6.3. MET and MHA Separation and Detection by UPLC-MS

Intracellular MET and MHA levels were measured simultaneously using a Waters^®^ Acquity H-Class Plus UHPLC System equipped with a thermostated autosampler and supported with a Waters^®^ single quadrupole mass detector QDa (Waters). The sample temperature was 6 °C. Chromatographic separation was achieved on a Phenomenex^®^ Luna Omega Polar C18 column (2.1 mm × 100 mm, i.d. 1.6 μm). The column was operated at 35 °C. The injection volume was 10 μL, and the flow rate was set at 0.4 mL/min. A ternary solvent system was used consisting of (A) pH 2.00 ± 0.02 ultrapure water, (B) acetonitrile and (C) ultrapure water. The mobile phase was filtered through in-line 0.2-μm membrane filters. The following gradient elution was employed: 0–2 min: 100% A; 2.1 min: 99% A, 1% B; 2.10–7 min: 99% A, 1% B; 7.1 min: 1% B, 99% C; 8 min: 60% B, 40% C; 8–11 min: 60% B, 40% C; 11.5 min: 100% A and 11.5–15 min: 100% A. The ionization source was used both in the electrospray (ESI)-negative and -positive modes, using single ion recording (SIR). The optimal cone voltage was set at 15 V and the capillary voltage at 0.5 kV; the source temperature was maintained at 600 °C. MET was monitored in ESI+ at 150 *m*/*z* in SIR mode. MHA was monitored in ESI- at 149 *m*/*z* in SIR mode.

#### 4.6.4. Biogenic Amino Acid Derivatization, Separation and Detection by HPLC-FL

Intracellular amino acid levels were performed using a Waters^®^ Alliance System (2695 separation module) equipped with a Waters^®^ 2695 Alliance autosampler and supported with a Waters^®^ 2475 Multi-λ Fluorescence Detector (Waters; Waters SAS Saint-Quentin-en-Yvelines, France). The investigated amino acids were cysteine (CYS), glutamic acid (GLU), glycine (GLY) and methionine (MET). Precolumn derivatization was done according to the instruction manual of the AccQTag kit (Waters^®^). Briefly, 60 μL of AccQ Fluor Borate buffer and 20 of μL AccQ Fluor derivatizing reagent were added to 20 µL of aqueous extracts with vortexing immediately and heating at 55 °C for 10 min. The samples were analyzed with an AccQTag 3.8-mm × 150-mm column. The flow rate was 1 mL/min, the injection volume was 20 μL, the column temperature was 37 °C and the run time was 50 min per sample. A ternary solvent system was used consisting of (A) pH 5.00 ± 0.05 20-mM phosphate buffer, (B) acetonitrile and (C) ultrapure water. The mobile phase was filtered through in-line 0.2-μm membrane filters. The gradient for the chromatographic separation was initially set to 100% A; then 99% A, 1% B from 0 to 0.5 min; then 95% A, 5% B from 0.5 to 18 min; then 91% A, 9% B from 18 to 19 min; then 83% A, 17% B from 19 to 29.5 min; then 60% B, 40% C from 29.5 to 33 min and then back to the initial conditions of 100% A at 36 min prior to equilibration until 50 min. The eluate was monitored under the following fluorescence parameters: 250 nm/395 nm (excitation/emission).

#### 4.6.5. Metabolites Quantification

Waters^®^ Empower™ Pro software was used for data acquisition. Metabolites were identified comparing their RT and *m*/*z* to the standard ones. The samples were quantified against standard curves of at least 6 points run in triplicate. Standard curves were run at the beginning and end of each chromatographic series. Quality control checks (blanks and standards) were run every 20 samples. Normalization was made using the total protein quantification obtained directly from the well using the bicinchoninic acid method, as previously described.

### 4.7. Statistical Analysis

For each experiment, the number of the biological replicates (N) was indicated in the figure legend, and all the values were presented as the means ± S.E.M. Normality was tested with the Shapiro–Wilk test for each condition independently (*p* > 0.05). Then, they were analyzed using a one-way ANOVA, with multiple comparisons performed with Tukey’s post hoc test. Conditions showing results statistically different from each other were presented using a different letter.

## Figures and Tables

**Table 1:** List of real-time quantitative PCR (RT-qPCR) primers used in this study.

Gene Name	Abbr.	Gene ID/Ref	Primers (5′→3′)	Eff.
*Elongation factor 1α*	*ef1α*	100136004 [13]	Fwd: TCCTCTTGGTCGTTTCGCTG Rev: ACCCGAGGGACATCCTGTG	1.91
*Asparagine synthetase*	*asns*	110488144 [31]	Fwd: CTGCACACGGTCTGGAGCTG Rev: GGATCTCGTCTGGGATCAGGTT	1.94
*DNA damage-inducible transcript 3*	*ddit3*	110494779 [32]	Fwd: CGACAATGTCCAACAACCTG Rev: ACGAGGAGAACGAGGTGCTA	1.97
*X-box binding protein 1*	*xbp1*	110526029 [31]	Fwd: TGCAACCAAGCCAATTCTTC Rev: GCGAGAACTTCGTCTTCCAG	1.95
*Monocarboxylate transporter 1-like*	*slc16a1-like*	110492722 [12]	Fwd: GAAGAAGGCGGAGTCTAATC Rev: TAGCGTAGTTGGAGAGGAA	2.04
*Monocarboxylate transporter 9-like*	*slc16a9-like*	110502533 [12]	Fwd: GTTGTTGGGTGGTTCTTTG Rev: GTCGATGTCAGCCTTCTT	1.97
*Monocarboxylate transporter 13*	*slc16a13*	110531725 [12]	Fwd: GTAGGCTATGCGTGAGTAAG Rev: GCCTCGAGCTAGTTGAATAA	1.88
*Sodium-coupled monocarboxylate transporter 1-like*	*slc5a8-like*	110494699 [12]	Fwd: GGCATCAGAACCTGAGATAA Rev: CAGTTGACAGAGTGCATTTAG	1.82
*methionine adenosyltransferase 2b*	*mat2b*	110533399 *	Fwd: GGCTCCAGGACCCATCAATA Rev: AGCTCAAGACGGGAACACTC	1.90
*methionine adenosyltransferase 2aa*	*mat2aa*	110509902 *	Fwd: GGCTATGACGACTCCTCCAA Rev: GCATAACCAAACATCAGACCCT	1.94
*methionine adenosyltransferase 2al*	*mat2al*	110537066 *	Fwd: ATCGGAGTCAGTTGGAGAGG Rev: TGACCTCTCCACACAGCAT	1.91
*DNA methyltransferase*	*dnmt1*	110486372 [33]	Fwd: TTGCCAGAAGAGGAGATGCC Rev: CCCAGGTCAGCTTGCCATTA	1.99
*adenosylhomocysteinase*	*ahcy*	110527644 [33]	Fwd: ATCAAACGGGCCACAGATGT Rev: TCGTACCTTCCATGGCAGC	1.94
*Cystathionine-beta-synthase*	*cbs1*	100136726 [33]	Fwd: CCACCTCAGGCAATACAGGT Rev: AACATCCACCTTCTCCATGC	1.98
*Cystathionine-beta-synthase*	*cbs2*	110520495 [33]	Fwd: CAAGGCTCTCAGCACATCCA Rev: ACCATCATCGAGCCCACCT	2.06
*Cystathionine gamma-lyase*	*cth1*	110524183 [33]	Fwd: CACCAACCCCACCATGAAAG Rev: GCGCTGGAAGTAGGCTGACA	1.95
*Cystathionine gamma-lyase*	*cth2*	110498361 *	Fwd: TGGCTTGAGACTCCCACCAA Rev: GCGCTGGAAGTAGGCTGACA	2.03
*glutamate-cysteine ligase catalytic subunit*	*gclc*	110499382 [34]	Fwd: CAACCAACTGGCAGACAATG Rev: CCTTTGACAAGGGGATGAGA	1.99
*5-methyltetrahydrofolate-homocysteine methyltransferase*	*mtr1*	110519474 [35]	Fwd: AATGCAGGTCTGCCCAATAC Rev: CTGATGTGTGCAGGAGTCGT	1.99
*5-methyltetrahydrofolate-homocysteine methyltransferase*	*mtr2*	110499554 *	Fwd: CCAGGAGTGTGGTGGTGTG Rev: CAGGAAGCGCCTCTCCTTTA	2.00
*Betaine-homocysteine methyltransferase*	*bhmt1*	110509664 [35]	Fwd: CAGAGAAGCACGGTAACTGG Rev: TTCTTTGTGCTGCATCAGGT	1.98
*Betaine-homocysteine methyltransferase*	*bhmt2*	110523446 [35]	Fwd: GCTGAGGAGCTAGCCACAGA Rev: GGCTTCAGCTTCTCCCAGTA	1.92
*glutathione synthetase*	*gss*	110532297 *	Fwd: TCAATACCATTGCTGCCAGTT Rev: ACTGCCCTTTCTGAGCCATA	1.93
*hydroxyacid oxidase 1*	*hao1*	110505655 *	Fwd: AGTTAGTGTGTGTGGCTGACT Rev: GACACATCCCTCAGTACCCT	1.94
*lactate dehydrogenase D*	*ldhd*	110494022 *	Fwd: GGCCGACATTCTGATCTGTG Rev: GACTCGTCTCTGCCATGTTG	2.00
*D-amino acid oxidase*	*dao*	110536118 *	Fwd: CGTTTGACTACCTGCTGAGC Rev: TCCACCATGAGAGCAGTGTT	1.98

Abbr.: Abbreviations; Gene ID: LOC number in assembly (NCBI, USDA_OmykA_1.1); Ref: References to primers already validated; *: primers were designed using Primer 3 software and validated for the study (verification of amplicon sizes through migration on agarose gel and sequencing). Eff: efficiency values determined upon the qPCR conditions described above.

## Data Availability

Data are available upon request to corresponding authors.

## Supplementary Materials

The following supporting information can be downloaded at https://www.mdpi.com/article/10.3390/ijms23062935/s1.
