# Supplementary material for: Are the Main Methionine Sources Equivalent? A Focus on DL-Methionine and DL-Methionine Hydroxy Analog Reveals Differences on Rainbow Trout Hepatic Cell Lines Functions
Source: Int J Mol Sci. 2022 Mar 8;23(6):2935. doi: 10.3390/ijms23062935 (PMC8954868; doi:10.3390/ijms23062935)
Supplement: Supplementary file 1 [file ijms-23-02935-s001.zip › ijms-1565898-supplementary.pdf]

Are The Main Methionine Sources Equivalent For Fish Nutrition? A Focus On DL-Methionine (DL-MET) And Methionine Hydroxy Analog (MHA-Ca) Reveals Differences On Rainbow Trout Cell Lines Functions.

Karine Pinel[1], Cécile Heraud[1], Guillaume Morin[1], Karine Dias[1], Annaëlle Marcé[1], Linda Beauclair[1], Stéphanie Fontagné-Dicharry[1], Karthik Masagounder[2], Martina Klüenemann[2], Iban Seiliez[1] and Florian Beaumatin [1,*]

Supplementary figures

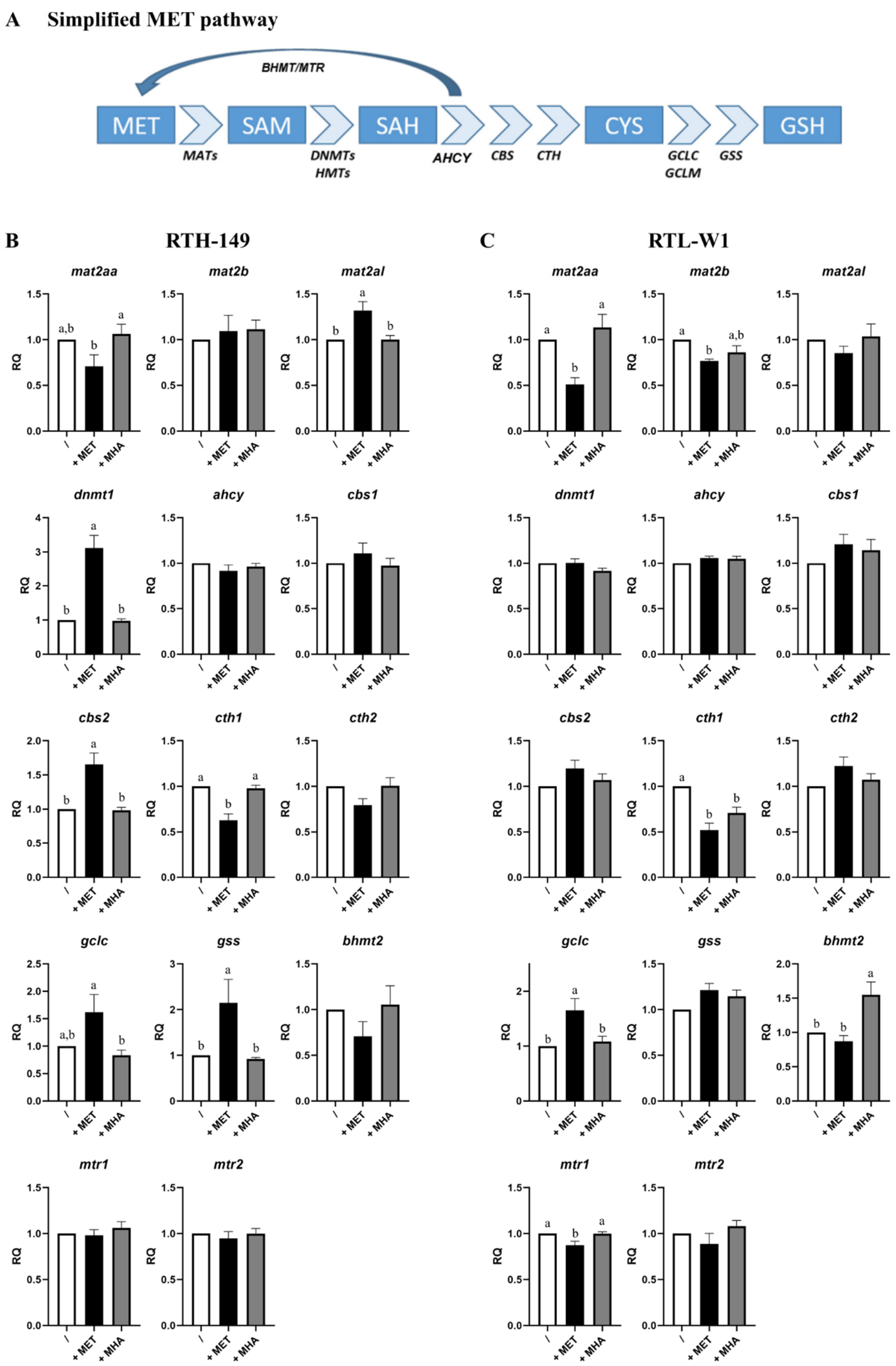

Supplementary Figure S1 Impact of MET sources on the expression of methionine-related metabolism genes.

(A) Diagram showing a simplified methionine cycle, transsulfuration pathway and glutathione (GSH) synthesis. Abbreviations: MET, methionine; MAT, methionine adenosyltransferase; SAM, S-adenosyl L methionine; DNMT1, DNA methyltransferase 1, SAH, S-adenosylhomocysteine; AHCY, adenosylhomocysteinase (also known as SAHH for S-adenosylhomocysteine-hydrolase; BHMT, betaine-homocysteine S-methyltransferase; MTR, 5-methyltetrahydrofolate-homocysteine methyltransferase; CBS, cystathionine-beta-synthase; CTH, cystathionine gamma-lyase; CYS, cysteine; GCLC, glutamate-cysteine lyase catalytic subunit; GCLM, glutamate-cysteine ligase modifier subunit; GSS, glutathione synthetase; GSH, glutathione. (B, C) Methionine cycle analysis with gene expression assessed by RT-qPCR following treatments with MET-depleted media (/) supplemented with MET (+MET) or MHA (+MHA) for RTH-149 cells (B) (N=7) or RTL-W1 cells (C) (N=5). Conditions showing results statistically different from each other are presented using a different letter (one-way ANOVA with Tukey's post-hoc test).

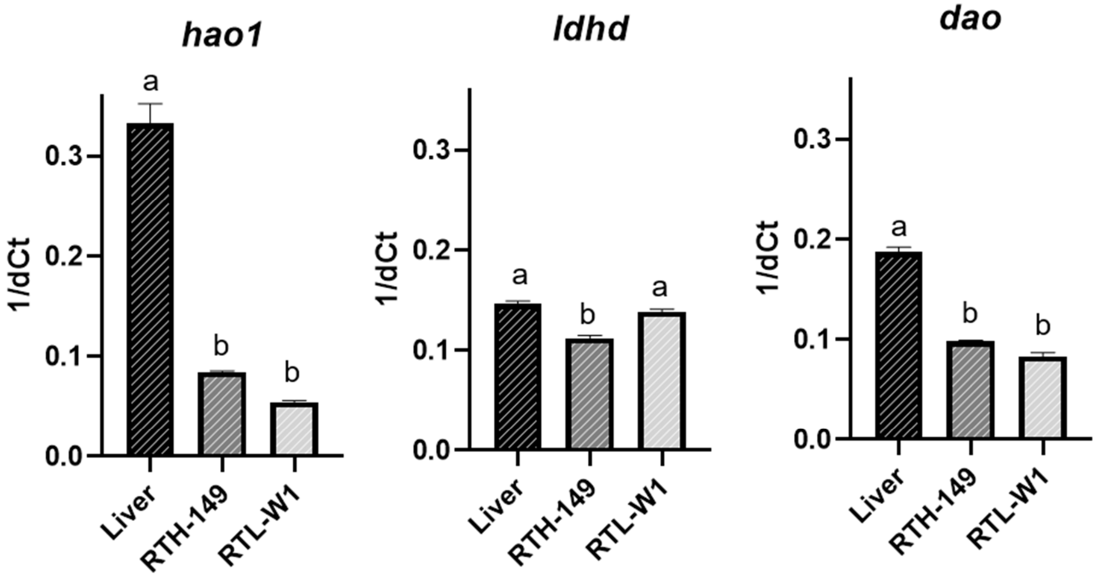

Supplementary Figure S2 Impact of MET sources on gene expression for the oxidation reactions according to MET sources considered.

Hydroxyacid oxidase 1 (hao1), lactate dehydrogenase d (ldhd) and d-amino acid oxidase (dao) gene expression were assessed by RT-qPCR for both RTH-149 (N=3) and RTL-W1 (N=3) cell lines compared as in liver tissues (N=9) respectively. Conditions showing results statistically different from each other are presented using a different letter (one-way ANOVA with Tukey's post-hoc test).
